# Circulating secretoneurin level reflects angiographic coronary collateralization in stable angina patients with chronic total occlusion

**DOI:** 10.1186/s12872-023-03645-6

**Published:** 2024-01-06

**Authors:** Zhi Ming Wu, Ke Huang, Yang Dai, Shuai Chen, Xiao Qun Wang, Chen Die Yang, Le Ying Li, Jing Meng Liu, Lin Lu, Rui Yan Zhang, Wei Feng Shen, Ying Shen, Feng Hua Ding

**Affiliations:** 1 Department of Cardiovascular Medicine, Rui Jin Hospital, Shanghai Jiao Tong University School of Medicine, 197 Rui Jin Road II, Shanghai, 200025 P.R. China; 2 Shanghai Clinical Research Center for Interventional Medicine, Shanghai, 200032 P.R. China

**Keywords:** Secretoneurin, Coronary collaterals, Chronic total occlusion, Stable angina, Neuropeptide

## Abstract

**Objective:**

To investigate the association between circulating secretoneurin (SN) and angiographic coronary collateralization in stable angina patients with chronic coronary total occlusion (CTO).

**Methods:**

SN concentrations in serum were measured in 641 stable angina patients with CTO by radioimmunoassay. The status of coronary collaterals from the contra-lateral vessel was visually estimated using the Rentrop grading system, and was categorized into poor (grade 0 or 1) or good (grade 2 or 3) collateralization.

**Results:**

Serum SN levels were significantly higher in patients with good coronary collaterals compared to those with poor collaterals (175.23 ± 52.09 pmol/L vs. 143.29 ± 42.01 pmol/L, *P* < 0.001). Serum SN increased stepwise across Rentrop score 0 to 3 (*P* < 0.001), and increasing SN tertiles were associated with higher proportion of good coronary collateralization (OR, 1.907; 95% CI, 1.558 ~ 2.335, *P* < 0.001). After adjustment for confounding variables, serum SN (per tertile) remained an independent factor for predicting good coronary collaterals (OR, 1.870; 95% CI, 1.515 ~ 2.309; *P* < 0.001). Moreover, the diagnostic value of serum SN (per tertile) was consistent after stratifying patients based on gender, age, body mass index, hypertension, diabetes, history of smoking, severity of coronary artery disease and kidney function (OR: 1.511 ~ 2.680, *P* interaction ≥ 0.327).

**Conclusion:**

Elevated circulating SN reflects good angiographic coronary collaterals in stable angina patients with CTO. The findings may provide insight into decision-making for these patients.

## Introduction

Coronary collateral vessels provide salvage to the myocardial areas subtended by severely stenotic or occluded epicardial vessels [1], and robust coronary collaterals are frequently correlated with smaller infarct size, better left ventricular function and lower mortality [2, 3]. In stable angina patients with chronic coronary total occlusion (CTO), a pressure gradient develops across the occluded bed, which increases blood flow in the preexisting arterioles and finally leads to structural augmentation (i.e. arteriogenesis) [4]. Although angiogenesis defined as a new capillary sprouting from existing vessels may provide some relief to ischemic or infarcted areas, arteriogenesis plays a main role in restoring blood supply after arterial occlusion [5]. Previous studies have shown that the development of coronary collaterals was highly influenced by individual clinical characteristics and multiple biochemical factors [6–8], and interestingly, patients with CTO often have a great deal of variability in the formation of coronary collateral vessels [9, 10].

Secretoneurin (SN) is produced and secreted by numerous cells throughout the body including myocardial cells, and has potential functions [11–13]. Several studies have demonstrated that this neuropeptide could serve as a novel biomarker of cardiovascular disease and predict prognosis in patients with acute and critical illness conditions [14, 15]. In animal experiments, SN induces coronary angiogenesis and helps to avoid ischemia-reperfusion injury and cardiomyocyte apoptosis via activating vascular endothelial growth factor pathway in endothelial cells in vitro and improving blood flow in vivo [16, 17]. However, data are still lacking on the association between serum SN and coronary collateral formation. In this study, we sought to investigate whether circulating SN reflects angiographic coronary collateralization in stable angina patients with CTO.

## Methods

The protocol was approved by the Institutional Review Board of Rui Jin Hospital, Shanghai Jiao Tong University School of Medicine. We obtained written informed consents from all patients and carried out the study following the Helsinki declaration.

### Study population

From the database of Shanghai Rui Jin Hospital Percutaneous Coronary Intervention (PCI) Outcome Program, consecutive 750 stable angina patients with at least one CTO lesion between December 2018 and September 2021 were recruited. Stable angina was defined according to the criteria formulated by the American College of Cardiology/American Heart Association [18]. CTO was defined as total occlusion for more than 3 months. Baseline demographic profiles, risk factors for coronary artery disease, biochemical measurements and medications were recorded. Hypertension, type 2 diabetes and dyslipidemia were diagnosed according to the JNC-7 criteria [19], the American Diabetes Association [20], and the National Cholesterol Education Program (ATP III) [21], respectively.

**Fig. 1:**
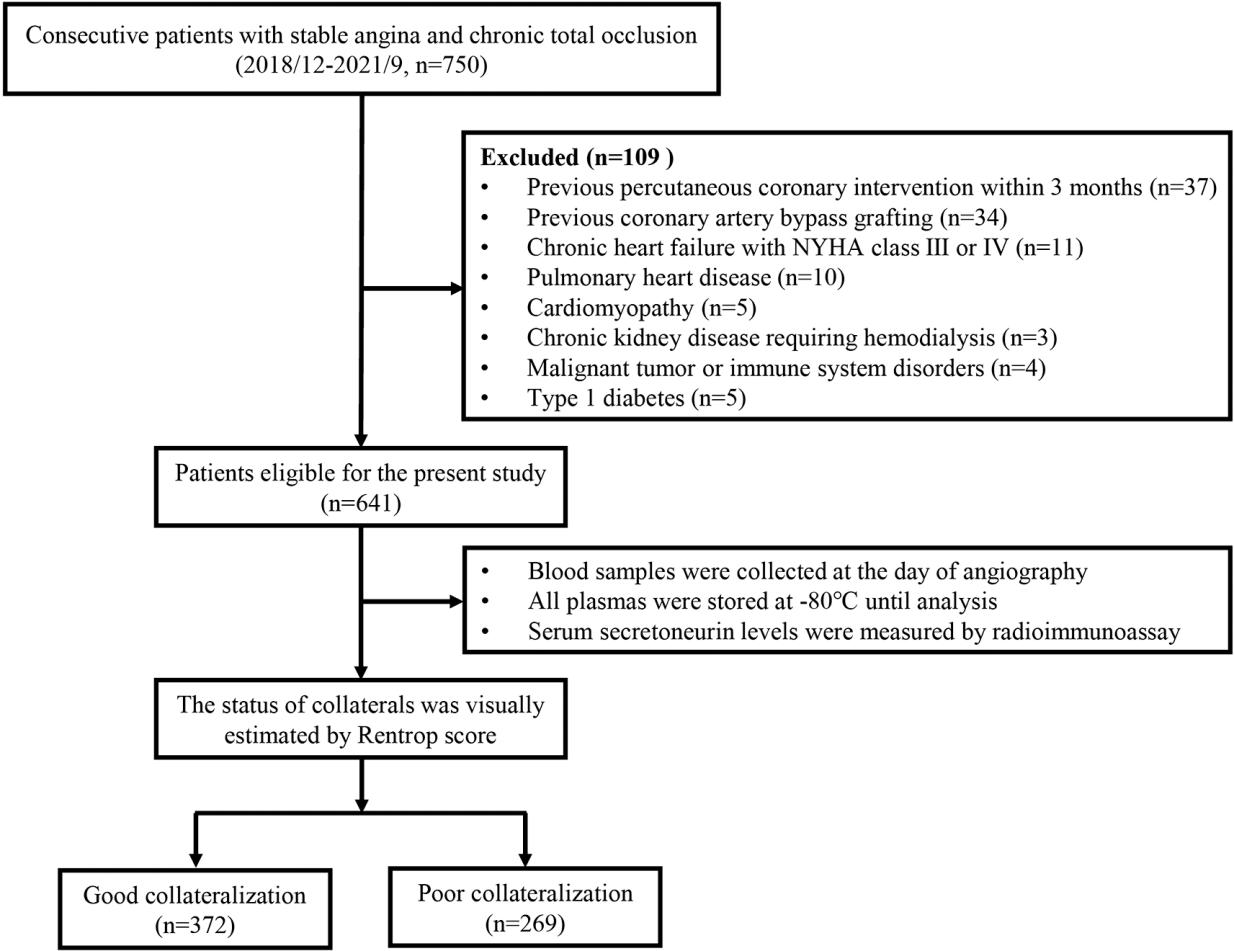
Flowchart of patient enrollment

For the purpose of this study, we excluded 109 patients with previous percutaneous coronary intervention within 3 months (n = 37), previous coronary artery bypass grafting (n = 34), NYHA class III-IV heart failure (n = 11), pulmonary heart disease (n = 10), cardiomyopathy (n = 5), chronic renal failure requiring hemodialysis (n = 3), malignant tumor or immune system disorders (n = 4) and type 1 diabetes by peptide C measurement (n = 5). Thus, a total of 641 eligible patients were included in the final analysis (Fig. 1). In addition, 52 coronary artery disease patients without CTO served as controls.

### Coronary angiography and collateral scoring

Standard selective coronary angiography was performed via the radial or femoral artery approach. Two interventional cardiologists who were blinded to biochemical results quantitatively analyzed all angiographic images via the Cardiovascular Measurement System software (version 3.0, USA). Number of significant diseased coronary arteries (≥ 50% lumen diameter narrowing in major epicardial coronary artery) was used to grade coronary artery disease severity, and left main coronary stenosis was regarded as 2-vessel disease.

J-CTO score was used to assess lesion difficulty during CTO PCI [22]. If a patient had more than one J-CTO score, the average value was calculated. Collaterals supplying to the segment distal to the occlusion from the contra-lateral vessel were visually estimated using the Rentrop score [23], as follows: 0 = no filling; 1 = filling of side branches only; 2 = partial filling of the epicardial vessel distal to the occlusion; 3 = adequate filling of the epicardial vessel distal to the occlusion by collaterals. Patients were categorized into poor (grade 0 or 1) or good (grade 2 or 3) coronary collateralization group according to the Rentrop score, as in our previous study [10, 24]. Analysis was conducted on the vessel with the highest Rentrop score if there was more than one totally occluded vessel. Any discordance was adjudicated by a third researcher.

### Biochemical measurement

Following an overnight fast, a blood sample was collected from each patient at the day of coronary angiography. To balance the fasting interval and avoid diurnal variation in SN level, we collected all blood samples at 6:00 am. Serum levels of glucose, uric acid, creatinine, blood urea nitrogen, and lipid profiles were automatically measured by standard techniques (HITACHI 912 Analyzer, Roche Diagnostics, Germany). Blood concentration of glycosylated hemoglobin (HbA1c) was determined by using ion-exchange high performance liquid chromatography with a Bio-rad Variant Hemoglobin Testing System (Bio-Rad Laboratories, Hercules, CA, USA). Serum level of high-sensitivity C-reactive protein (hsCRP) was assessed by ELISA (Biocheck Laboratories, Toledo, OH, USA). Glomerular filtration rate (GFR) was calculated by CKD-EPI formula [25].

For gauging the concentration of SN, each blood sample was centrifuged at 1500 g for 10 min and the resulting serum samples were aliquoted and transferred to -80 °C until assessment. We determined serum SN concentration by radioimmunoassay in automatic gamma counter following the protocols according to previous studies [12, 14].

### Statistical analysis

Data are presented as mean and standard deviation (SD) or median (interquartile range [IQR]) for continuous variables, and as absolute numbers with percentages for categorical variables. We used Chi-square tests for comparing the differences in categorical variables, and chose student t-tests or one-way analysis of variance to compare the difference of normally distributed continuous variables; otherwise, Mann-Whitney U tests were chosen. Spearman’s rho tests were chosen to determine the correlation between SN and Rentrop score. For assessing the independent determinants of good collateralization, clinical characteristics and biochemical and angiographic results including gender, age, body mass index (BMI), traditional risk factors (hypertension, diabetes, smoking), number of diseased coronary arteries, GFR, total-to-high-density lipoprotein (HDL) cholesterol ratio, hsCRP were adopted in model 1 and along with SN in model 2 for multivariate logistic regression analyses. We used predicted probabilities for good coronary collaterals derived from two logistic regression models above to perform receiver operating characteristic (ROC) analysis and chose DeLong method to compare the areas under the curve by MedCalc software (version 19.8, Ostend, Belgium). All tests were 2-sides and made using SPSS (version 25.0, USA) with an overall significance alpha level of 0.05.

## Results

### Baseline characteristics

Among 641 patients with CTO, 372 (58.0%) had good coronary collaterals and 269 had poor coronary collaterals. Diabetes and cigarette smoking were less common, HbA1c and hsCRP levels were lower, but the proportions of male and hypertension were higher in good collateralization group (*P* < 0.05 for all comparisons). Coronary artery disease severity and medications were not significantly different between the two groups (Table 1). There were no significant differences with respect to age, sex, proportion of hypertension, dyslipidemia and smoking between CTO and non-CTO patients. However, proportion of diabetes and severity of coronary artery disease were lower in patients without CTO. All non-CTO patients had no angiographic coronary collaterals.

**Table 1:** Baseline demographic and clinical characteristics in patients with poor and good collateralization

	Poor collateralization (n = 269)	Good collateralization (n = 372)	*P* value
Male, n (%)	204 (75.8)	306 (82.3)	0.047
Age, years	64.98 ± 9.20	63.95 ± 9.26	0.167
Body mass index, Kg/m^2^	25.29 ± 3.60	24.80 ± 3.20	0.072
Systolic blood pressure, mm Hg	133.57 ± 21.72	135.41 ± 19.70	0.263
Diastolic blood pressure, mm Hg	75.19 ± 11.94	75.48 ± 11.96	0.761
Hypertension, n (%)	173 (64.3)	267 (71.8)	0.044
Diabetes, n (%)	158 (58.7)	167 (44.9)	0.001
Cigarette smoking, n (%)	106 (39.4)	110 (29.6)	0.009
Dyslipidemia, n (%)	77 (28.6)	88 (23.7)	0.156
Fasting blood glucose, mmol/L	6.51 ± 2.61	6.30 ± 2.16	0.269
Serum uric acid, µmol/L	372.57 ± 114.04	365.21 ± 102.26	0.392
Serum creatinine, µmol/L	93.04 ± 57.23	92.20 ± 73.12	0.875
BUN, mmol/L	6.74 ± 3.94	6.39 ± 2.58	0.199
HbA1c, %	6.61 ± 1.43	6.36 ± 1.20	0.020
GFR, mL/min/1.73m^2^	77.38 ± 20.70	79.88 ± 18.88	0.112
Triglyceride, mmol/L	1.78 ± 1.31	1.75 ± 1.33	0.808
Total cholesterol, mmol/L	4.23 ± 1.18	4.18 ± 1.17	0.593
HDL cholesterol, mmol/L	1.03 ± 0.24	1.06 ± 0.25	0.238
LDL cholesterol, mmol/L	2.59 ± 0.99	2.54 ± 1.05	0.575
Apolipoprotein A, g/L	1.13 ± 0.20	1.15 ± 0.21	0.179
Apolipoprotein B, g/L	0.85 ± 0.26	0.84 ± 0.26	0.694
Lipoprotein (a), g/L	0.32 ± 0.57	0.30 ± 0.41	0.514
hsCRP, mg/L	2.12 (0.76 ~ 8.00)	1.33 (0.59 ~ 5.61)	0.001
Severity of CAD, n (%)			
1-vessel	36 (13.4)	46 (12.4)	0.704
2-vessel	62 (23.0)	96 (25.8)	0.424
3-vessel	171 (63.6)	230 (61.8)	0.653
Peripheral artery disease, n (%)			
Carotid artery stenosis ≥ 50%	34 (12.6)	41 (11.0)	0.529
Arteriosclerosis obliterans of lower extremities	17 (6.3)	19 (5.1)	0.511
Medication, n (%)			
ACE inhibitors/ARBs	160 (59.5)	226 (60.8)	0.745
β-blockers	125 (46.5)	185 (49.7)	0.415
Calcium channel blockers	66 (24.5)	101 (27.2)	0.457
Nitrates	113 (42.0)	159 (42.7)	0.853
Diuretic	65 (24.2)	71 (19.1)	0.121
Statins	189 (70.3)	278 (74.7)	0.209
Secretoneurin (pmol/L)	143.29 ± 42.01	175.23 ± 52.09	< 0.001

Values are given as mean ± standard deviation (SD), median (25th ~ 75th percentile) or number (percentage)

ACE, angiotensin converting enzyme; ARB, angiotensin receptor blocker; BUN, blood urea nitrogen; CAD, coronary artery disease; GFR, glomerular filtration rate; HbA1c, glycated hemoglobin; HDL, high-density lipoprotein; hsCRP, high-sensitivity C-reactive protein; LDL, low-density lipoprotein

### Serum SN and collateral formation

Serum SN levels were significantly lower in non-CTO than in CTO patients (103.26 ± 12.63 vs. 161.82 ± 50.60 pmol/L, *P* < 0.001). For patients with CTO, serum SN was significantly higher in patients with good coronary collaterals than those with poor collaterals (175.23 ± 52.09 vs. 143.29 ± 42.01 pmol/L, *P* < 0.001) (Table 1).

In overall 641 patients with CTO, 608 (94.9%) had right coronary dominance. A total of 717 CTO lesions were detected, among which 244 (34.0%) were presented in left anterior descending artery (LAD), 120 (16.7%) in left circumflex artery (LCX), and 353 (49.2%) in right coronary artery (RCA). The number of CTO occurring at ostial, proximal, middle and distal segment of the coronary artery was 76 (10.6%), 304 (42.4%), 227 (31.7%), and 66 (9.2%), respectively. Sixteen (2.2%) and 28 (3.9%) CTO lesions were located at posterior descending or posterolateral artery, respectively. No significant differences in SN levels were observed according to coronary dominance (Right dominance: 161.94 ± 50.69; Left dominance: 159.62 ± 49.71 pmol/L, *P* = 0.797), occluded vessel (LAD: 165.95 ± 51.76; LCX: 156.43 ± 43.05; RCA: 160.42 ± 51.43 pmol/L, *P* = 0.167) and site of CTO (ostial: 157.57 ± 51.76; proximal: 162.90 ± 48.70; middle: 161.36 ± 50.65; distal: 162.74 ± 54.62; posterior descending, 156.16 ± 50.92; posterolateral: 161.59 ± 53.20 pmol/L, *P* = 0.969), respectively. Similarly, there was no significant difference in SN level between CTO patients with in-stent restenosis (n = 46) and those with de novo lesions (n = 595) (156.65 ± 53.86 vs. 162.22 ± 50.37 pmol/L, *P* = 0.472). No differences in serum SN existed across patients with J-CTO score from 0 to 5 (0: 164.91 ± 50.43; 1: 162.74 ± 49.68; 2: 161.08 ± 49.07; 3: 161.50 ± 53.64; 4: 161.89 ± 50.15; 5: 163.19 ± 21.70 pmol/L, *P* = 0.999). In 433 (67.6%) patients who underwent CTO PCI, the technical success was achieved in 391 (90.3%) patients. Serum SN level was similar in patients with or without successful CTO PCI (162.21 ± 50.27 vs. 159.23 ± 55.91 pmol/L, *P* = 0.718).

**Fig. 2:**
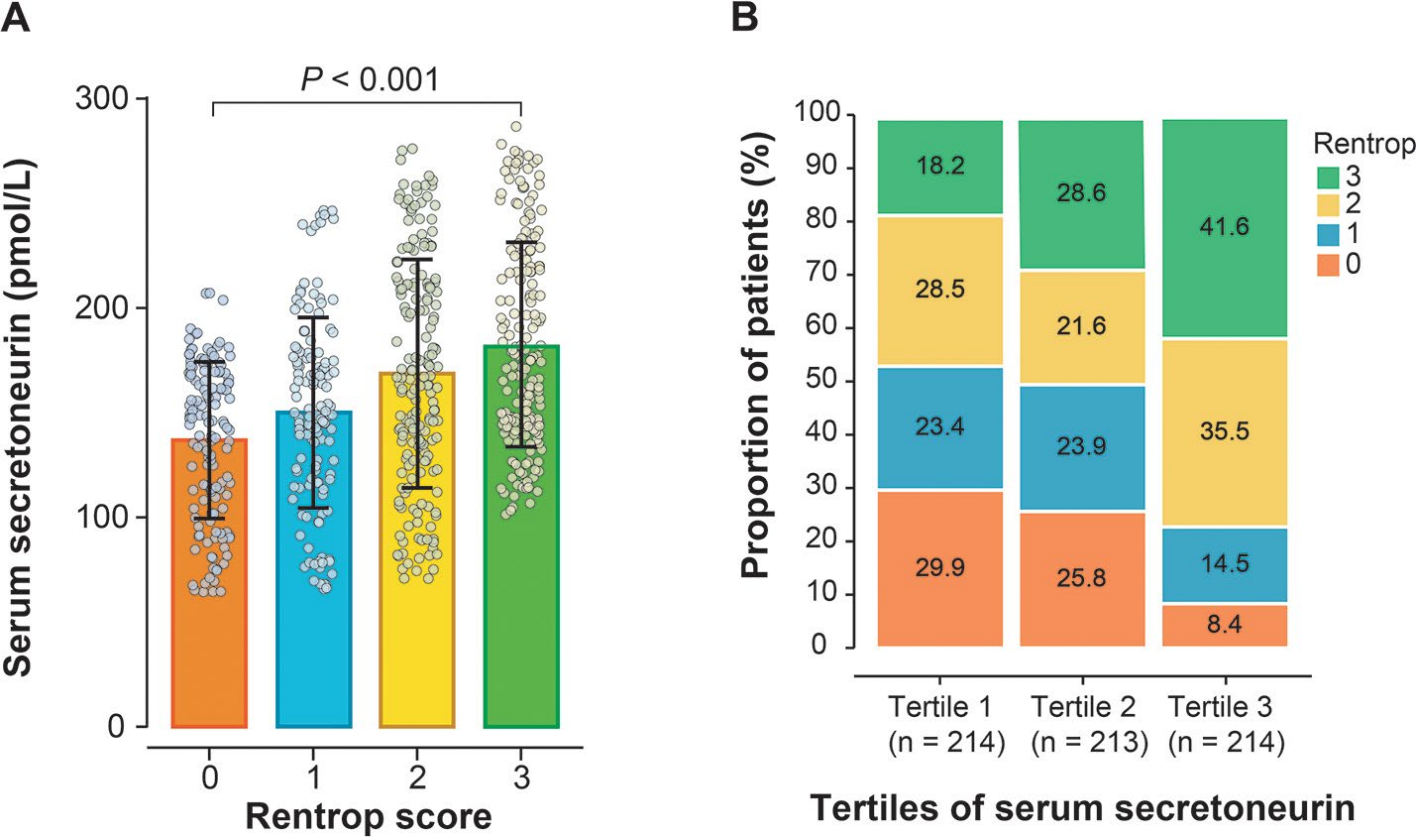
Relationship between serum SN and Rentrop score. (**A**) Serum SN increased gradually with Rentrop score. (**B**) The proportion of good coronary collateral formation increased stepwise from the lowest tertile to the highest tertile of SN

**Fig. 3:**
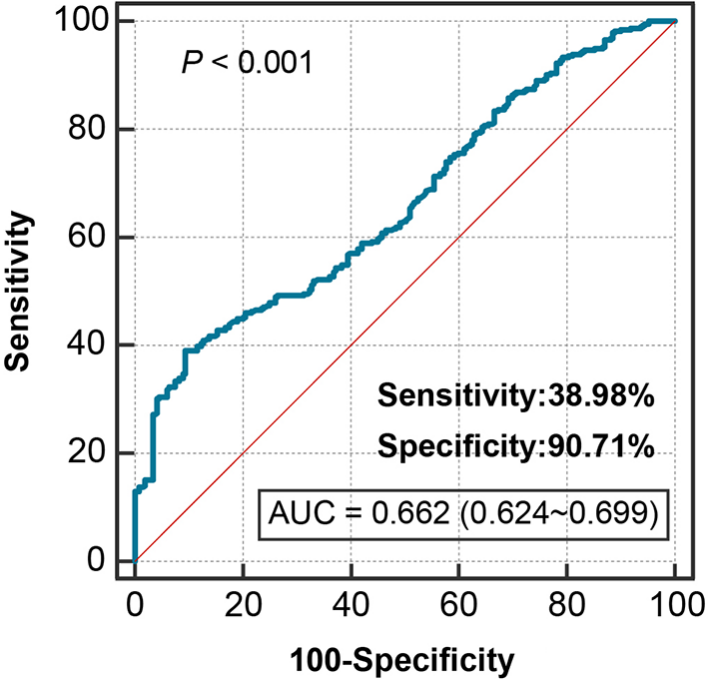
Receiver operating characteristic curve analysis of detecting good collateralization. An optimal cutoff of serum SN 190.12 pmol/L predicted good coronary collateralization (AUC 0.662, 95% CI, 0.624 ~ 0.699; *P*＜0.001; sensitivity 38.98% and specificity 90.71%)

Serum SN increased stepwise across Rentrop score 0 to 3 (*P* < 0.001) (Fig. 2A), and increasing SN tertiles were associated with higher proportion of good coronary collateralization (odds ratio [OR], 1.907; 95% confidence interval [CI], 1.558 ~ 2.335, *P* < 0.001) (Fig. 2B). After adjustment for gender, age, BMI, traditional risk factors for coronary artery disease, number of diseased coronary arteries, GFR, total-to-HDL cholesterol ratio, and hsCRP, serum SN level retained a positive correlation with Rentrop score (adjusted Spearmen’s r = 0.319, *P* < 0.001). In ROC curve analysis, the optimal cutoff value of serum SN for predicting good coronary collaterals was 190.12 pmol/L, with 38.98% sensitivity and 90.71% specificity [area under the curve 0.662 (95% CI, 0.624 ~ 0.699); *P* < 0.001] (Fig. 3).

Multivariate logistic regression analyses revealed that male, history of hypertension and diabetes, smoking, and hsCRP were significantly related to coronary collaterals (model 1). Serum SN (per tertile) remained an independent factor of good coronary collaterals after adjustment for all variables in model 1 (OR, 1.870; 95% CI, 1.515 ~ 2.309; *P* < 0.001) (Table 2). Inclusion of SN had improved goodness of fit with increased Nagelkerke R square by 6.7% (16.4% vs. 9.7%, *P* < 0.001) and increased C statistic by 0.057 (95% CI, 0.026 ~ 0.088, *P* < 0.001) (model 2).

**Table 2:** Multivariate logistic regression analyses for good coronary collaterals in patients with chronic total occlusion

	Variables	OR (95% CI)	*P* value
**Model 1**	Male	1.680 (1.109 ~ 2.545)	0.014
	Age (per 10 years)	0.861 (0.709 ~ 1.044)	0.127
	Body mass index (per SD)	0.880 (0.747 ~ 1.035)	0.123
	History of hypertension	1.518 (1.065 ~ 2.162)	0.021
	History of diabetes	0.552 (0.397 ~ 0.768)	< 0.001
	Smoking	0.521 (0.362 ~ 0.750)	< 0.001
	Severity of coronary artery disease	0.996 (0.790 ~ 1.255)	0.971
	GFR (per SD)	1.152 (0.966 ~ 1.374)	0.114
	Total-to-HDL cholesterol ratio (per SD)	0.890 (0.755 ~ 1.050)	0.167
	hsCRP (per SD)	0.821 (0.686 ~ 0.982)	0.031
**Model 2**	Male	1.620 (1.056 ~ 2.486)	0.027
	Age (per 10 years)	0.882 (0.724 ~ 1.075)	0.213
	Body mass index (per SD)	0.855 (0.724 ~ 1.010)	0.066
	History of hypertension	1.511 (1.049 ~ 2.176)	0.027
	History of diabetes	0.576 (0.410 ~ 0.809)	0.001
	Smoking	0.556 (0.383 ~ 0.808)	0.002
	Severity of coronary artery disease	0.981 (0.774 ~ 1.243)	0.872
	GFR (per SD)	1.140 (0.950 ~ 1.367)	0.158
	Total-to-HDL cholesterol ratio (per SD)	0.901 (0.760 ~ 1.069)	0.233
	hsCRP (per SD)	0.809 (0.671 ~ 0.976)	0.027
	Tertiles of secretoneurin	1.870 (1.515 ~ 2.309)	< 0.001

Values are odds ratios (95% confidence interval)

CI, confidence interval; HDL, high-density lipoprotein; GFR, glomerular filtration rate; hsCRP, high-sensitivity C-reactive protein; OR, odds ratio; SD, standard deviation

### Diagnostic value of SN in subgroup analysis

**Fig. 4:**
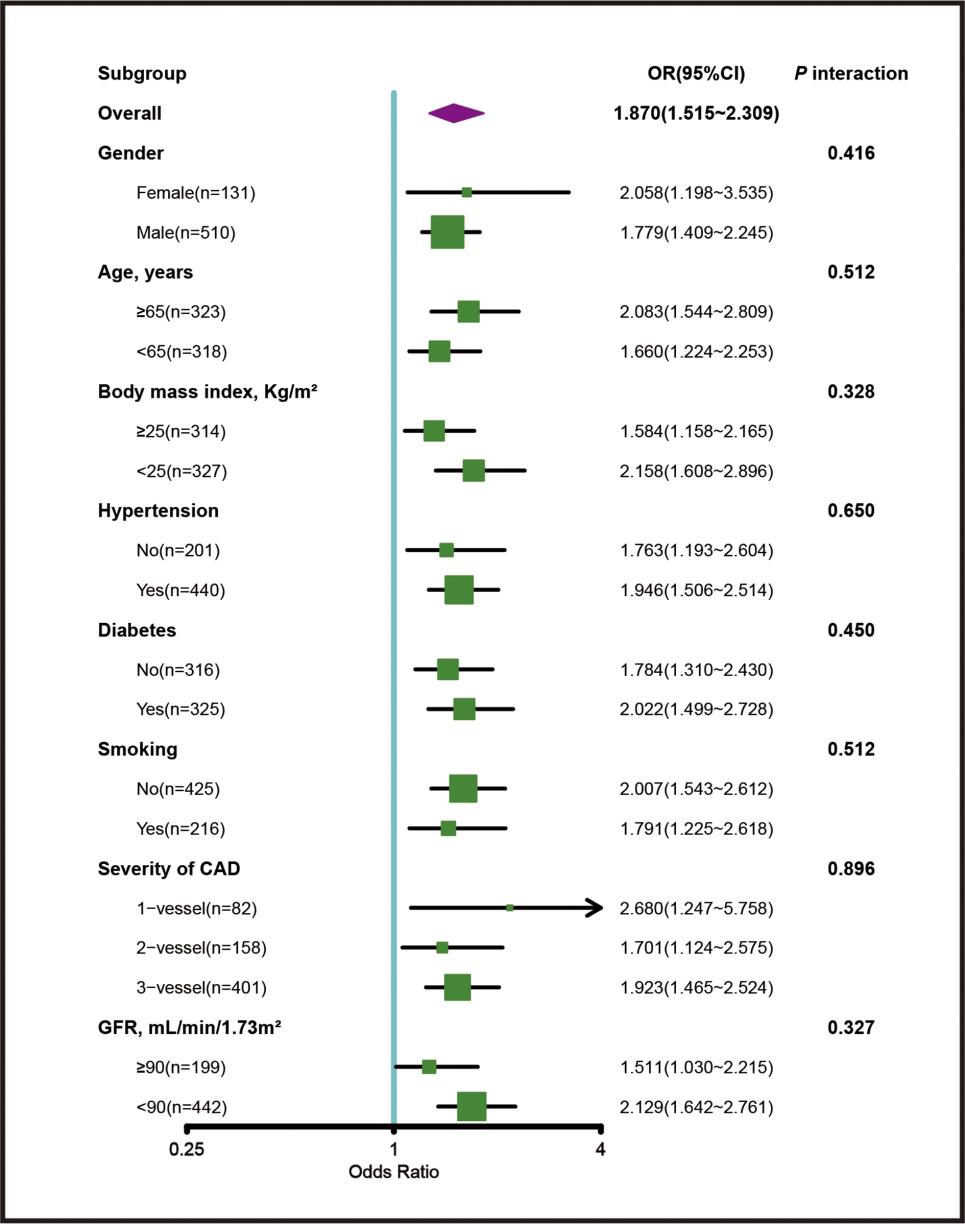
Diagnostic value of SN in patient subgroup analysis. CAD, coronary artery disease; CI, confidence interval; GFR, glomerular filtration rate; OR, odds ratio

The sensitivity analyses were conducted in subgroups stratified by gender, age, BMI, hypertension, diabetes, smoking, number of diseased coronary arteries and kidney function. There was a consistent diagnostic value of SN (per tertile) for detecting good coronary collaterals with OR ranging from 1.511 to 2.680 (*P* interaction ≥ 0.327) (Fig. 4).

## Discussion

Our results revealed that elevated serum SN was associated with good angiographic coronary collaterals in stable angina patients with CTO. These observations may be clinically relevant for the management of stable coronary artery disease.

It is well recognized that the growth and maturation of collateral vessels are simultaneously governed by pro- and anti-angiogenic factors [4, 7]. SN is an important member of the chromogranin family [26]. It acts as a potent pro-angiogenic factor and induces angiogenesis, arteriogenesis, and vasculogenesis [27]. To better characterize the role of SN in coronary collateralization, we selected a unique study population, as all patients had stable angina and total occlusion of at least one major epicardial coronary artery. The major finding is that there existed a close correlation between serum SN and Rentrop score in these patients, even after adjustment for various factors. Furthermore, the diagnostic performance of serum SN for coronary collateral status was equally good in different subgroups of patients stratified according to clinical profiles, coronary disease severity and kidney function. These observations support a notion that circulating SN may represent a biomarker reflecting coronary collateral formation and complementing established risk indices in patients with stable angina.

The association of serum SN with coronary collateralization may be, at least partially, the result of its net pathophysiological effects on hypoxia-driven induction of neo-vascularization in ischemic diseases. Nerves are proved to be involved in regulation of vessel growth as well as remodeling via release of numerous neuropeptides and neurotransmitters. It was reported that SN resides in nerve fibers with close blood vessel interactions [28]. In vivo, collateral vessel’s adventitia contains abundant SN-immunoreactive nerve fibers. Vascular smooth muscle cells could be stimulated to proliferate by SN, transforming small arterioles into more mature collaterals [27]. Kirchmair et al. found that this neuropeptide also plays a directly angiogenic role in vitro and in vivo, which increased circulating endothelial progenitor cell number and exerted antiapoptotic effects [29]. In addition, SN induced coronary angiogenesis by activating vascular endothelial growth factor pathway, enhanced blood recovery [17, 30, 31], and improved ventricular function [16]. Recently, Liu et al. reported that SN could act as a mediator of inflammation, and administration of SN promoted infiltration of inflammatory cells/macrophages in the vascular wall and enhanced arteriogenesis and collateral formation reflected by higher arteriole/artery density and a mature collateral network [27].

The findings of our study may have certain clinical relevance. A positive relation between elevated circulating SN and good coronary collateralization may underscore its putative use as a biomarker of collateral status in patients who have severe coronary obstruction or total occlusion. Emerging evidence suggests that treatment decision-making and indication for recanalization of a CTO should be based not only on clinical characteristics and occluded lesion morphology, but also on collateral quality and myocardial viability [32]. Successful recanalization of a CTO with well-developed collaterals was related to better left ventricular functional recovery and long-term outcomes [33]. In addition, several animal model studies have shown that SN might qualify as a promising treatment. In fact, gene therapy with SN has been demonstrated to induce therapeutic angiogenesis via a nitric oxide-dependent mechanism, and benefit Apo E-/- mice following hind-limb ischemia without influencing atherosclerosis [17, 34]. Finally, our results will provide an impetus for further large-scale, prospective studies to investigate the prognostic role of SN with coronary collateral growth in patients with stable angina.

## Study limitations

We recognized several limitations in the present study. First, this is a cross-sectional study which allowed us to detect the association rather than formulate causal link between serum SN and collateral growth. However, the prescription data were precisely gained from standard database, thus our results could truly reflect real-world associations. Second, the time-line of CTO formation and collateral development was unclear, and angiographic presence of coronary collaterals did not allow an accurate statement about myocardial viability. While a positive association between angiographic collateralization and SN has been detected, the relationship between SN levels and the extent of myocardial ischemia remains unknown. In addition, it remains uncertain if this also reflects the desired positive prognostic value. Thus, a longitudinal study is needed to clarify the value of serum SN in predicting CTO or judging the prognosis. Finally, the usage of Rentrop score is an insufficient tool for the assessment of coronary collaterals because of its qualitative character and the dependence of several factors. Determination of collateral flow index may be more accurate in assessing coronary collateralization [3].

## Conclusions

This study is the first to indicate that elevated circulating SN level could reflect good angiographic coronary collateralization in patients with stable angina and CTO. Our results are hypothesis generating and provide new insights on the value of pro-angiogenic SN for assessing coronary collateral status in this setting. Further studies are warranted to determine if the association between serum SN and collateral formation could translate into incremental therapeutic and prognostic information to established risk indices in patients with chronic coronary syndrome.

## Data Availability

The datasets used and/or analyzed during the current study are available from the corresponding author on reasonable request.

## List of abbreviations

ACE: angiotensin converting enzyme
ARB: angiotensin receptor blocker
AUC: area under the curve
BMI: body mass index
BUN: blood urea nitrogen
CAD: coronary artery disease
CI: confidence interval
CTO: chronic coronary total occlusion
GFR: glomerular filtration rate
HbA1c: glycated hemoglobin
HDL: high-density lipoprotein
hsCRP: high-sensitivity C-reactive protein
IQR: interquartile range
LDL: low-density lipoprotein
OR: odds ratio
ROC: receiver operating characteristic
SD: standard deviation
SN: secretoneurin
